# Dermofunctional Vehicle Downregulates LL-37 and MMPs and Upregulates IGFBP-3

**DOI:** 10.3390/cimb48010054

**Published:** 2025-12-31

**Authors:** Hudson Polonini, Fabiana Regina da Silva Olímpio, Carlos Rocha Oliveira

**Affiliations:** 1 Fagron BV, Fascinatio Boulevard 350, 3065 WB Rotterdam, The Netherlands; hudson.polonini@fagron.com; 2 Gap Biotech, Biotechnology Laboratory, Sao Jose dos Campos 12243-020, Brazil; fabiana.olimpio@unifesp.br; 3 Postgraduate Program in Biomedical Engineering, Department of Science and Technology, Federal University of Sao Paulo (UNIFESP), Sao Jose dos Campos 12231-280, Brazil

**Keywords:** Cleoderm™, keratinocytes, fibroblasts, LL-37, IGFBP-3, IL-6, TNF-α, TGF-β, IL-10, MMP-1, MMP-3, MMP-13, dermocosmetic base, acne, rosacea

## Abstract

Background: Functional dermatological bases can contribute more than just delivery—they may actively modulate cutaneous homeostasis. Cleoderm™ is a dermofunctional base containing a patented *Cleome gynandra* extract, palmitoyl tripeptide-8, bisabolol, hyaluronic acid, and functional oils, rationally designed to provide anti-inflammatory, antioxidant, and barrier-supportive properties. Objective: To determine whether Cleoderm™ exhibits intrinsic immunomodulatory and matrix-protective effects in a physiologically relevant skin co-culture and to clarify the biomarkers most impacted, with translational relevance to acne and rosacea. Methods: Human keratinocytes and fibroblasts were maintained in a transwell co-culture. Non-cytotoxic concentrations of Cleoderm™ (1.0% and 10.0%, *v/v*) were tested with or without LPS stimulation (1 μg/mL). Viability was assessed by MTT and Trypan Blue. Cytokines (IL-6, TNF-α, IL-10, TGF-β) and MMPs (MMP-1, -3, -13) were quantified by ELISA and RT-qPCR. LL-37, IGFBP-3, and TGF-β protein levels were evaluated by Western blot. Results: Cleoderm™ showed no cytotoxicity up to 10% (*v/v*). It significantly reduced pro-inflammatory mediators (IL-6, TNF-α) and matrix-degrading enzymes (MMP-1, MMP-3, MMP-13) while increasing anti-inflammatory/reparative cytokines (IL-10, TGF-β). A dual, biomarker-level modulation was observed: (i) LL-37 was reduced, with a particularly pronounced decrease in secreted levels; and (ii) IGFBP-3 was markedly upregulated, indicating potential attenuation of the IGF-1 axis relevant to sebaceous lipogenesis. Collectively, these effects indicate immunoregulatory and matrix-protective activity consistent with improved cutaneous homeostasis. Conclusion: In a dermo-epidermally relevant in vitro model, Cleoderm™ functions as an active dermofunctional base, not merely a vehicle simultaneously tempering inflammatory signaling, preserving extracellular matrix integrity, and modulating mechanistic nodes (LL-37 and IGFBP-3) linked to rosacea and acne. These findings is consistent with the use of Cleoderm™ as a biologically supportive base for personalized compounding and justify controlled clinical evaluation.

## 1. Introduction

Dermatological diseases constitute a major global public health concern due to their high prevalence, chronic nature, and socioeconomic impact. It is estimated that between 30% and 70% of individuals worldwide experience at least one skin condition during their lifetime—for example, in the United States alone, one in three people is affected by dermatological diseases [1,2,3]. These numbers emphasize the need for therapeutic approaches that are both clinically effective and economically sustainable.

Among the most prevalent dermatoses are acne vulgaris, rosacea, seborrheic dermatitis, and post-inflammatory hyperpigmentation. Although these conditions differ in etiology, they share common pathogenic mechanisms such as chronic local inflammation, epidermal barrier disruption, oxidative stress, and dysregulation of sebum production. Beyond their biological impact, these diseases are strongly associated with psychosocial burden, including reduced self-esteem, anxiety, and impaired quality of life [4,5].

Topical therapy remains the cornerstone of treatment for most inflammatory skin disorders, offering localized efficacy and minimized systemic risk. In this context, the pharmaceutical vehicle is not merely an inert carrier but a decisive element in treatment success. The vehicle influences drug solubility, penetration, release kinetics, tolerability, and adherence—particularly in formulations for sensitive or compromised skin [6,7,8]. Consequently, the development of functional dermocosmetic bases capable of exerting intrinsic biological activity (anti-inflammatory, antioxidant, barrier-restoring, or microbiome-stabilizing) represents a significant advancement in personalized dermatology and pharmaceutical compounding.

Cleoderm™ was developed to meet this demand as a studied formulation dermofunctional base designed for customized dermatological formulations. Its composition combines a patented extract of *Cleome gynandra* (rich in polyphenolic antioxidants), palmitoyl tripeptide-8 (a neuro-soothing peptide with anti-inflammatory properties), bisabolol, hyaluronic acid, and a synergistic blend of functional oils including avocado, jojoba, rosehip, coconut, lavender, melaleuca, rosemary, shea butter, and vitamin E. Together, these components contribute complementary antioxidant, antimicrobial, barrier-repairing, and sebum-regulating properties supported by preclinical and clinical evidence [9,10,11,12,13,14]. Furthermore, Cleoderm™ exhibits an excellent safety and tolerability profile, including low comedogenicity, making it suitable for facial use and sensitive skin [15].

Beyond its role as a vehicle, Cleoderm™ was conceived as a biologically active platform capable of modulating cutaneous homeostasis. This aligns with growing interest in dermofunctional bases that not only facilitate drug delivery but also engage with biological pathways relevant to inflammation and skin repair. For example, cathelicidin LL-37 plays a critical role in rosacea by driving inflammation, vascular reactivity, and tissue damage, while the IGF-1/IGFBP axis regulates sebaceous lipogenesis and keratinocyte proliferation in acne pathophysiology [16,17,18,19]. Modulation of these pathways may enhance therapeutic outcomes, reduce the required concentrations of pharmacologically active agents, and minimize potential side effects.

To explore these mechanisms, this study employed a physiologically relevant transwell co-culture system of keratinocytes and fibroblasts [20]. Our primary hypothesis was that Cleoderm™ acts as an active dermofunctional base capable of modulating key pathogenic pathways, particularly those involved in chronic inflammation and sebaceous regulation. Using this model, we investigated Cleoderm™’s potential for intrinsic immunomodulatory effects that could support and enhance the efficacy of active ingredients in compounded formulations. We defined the modulation of LL-37 and IGFBP-3 as primary outcomes due to their direct link to the pathophysiology of rosacea and acne, respectively, supplemented by secondary outcomes related to inflammatory cytokines and MMPs [21]. By elucidating these properties, this work seeks to position Cleoderm™ as a functional dermocosmetic base that integrates pharmacological performance into personalized compounding practice.

## 2. Materials and Methods

### 2.1. Reagents

The investigational product Cleoderm™ (Fagron BV, Rotterdam, The Netherlands) was tested at concentrations ranging from 0.001% to 10% (*w/w*). For cell culture, we employed Dulbecco’s Modified Eagle Medium (DMEM) (Gibco, Carlsbad, CA, USA) supplemented with Fetal Bovine Serum (FBS) (Gibco, Carlsbad, CA, USA), L-glutamine (Gibco, Carlsbad, CA, USA), sodium bicarbonate (Gibco, Carlsbad, CA, USA), streptomycin (Sigma-Aldrich, St. Louis, MO, USA), penicillin (Sigma-Aldrich, St. Louis, MO, USA), and gentamicin (Sigma-Aldrich, St. Louis, MO, USA). For cytotoxicity assays, MTT reagent (3-(4,5-dimethylthiazol-2-yl)-2,5-diphenyltetrazolium bromide) (Sigma-Aldrich, St. Louis, MO, USA) and 0.05% Trypan Blue solution (Sigma-Aldrich, St. Louis, MO, USA) were used, with Dimethyl sulfoxide (DMSO) (Sigma-Aldrich, St. Louis, MO, USA) employed to dissolve the formed crystals in the MTT assay. Cell stimulation was performed using Lipopolysaccharide (LPS) (Sigma-Aldrich, St. Louis, MO, USA) at a concentration of 1 µg/mL. For Western blot analysis, the protein quantification was performed using the Bradford assay kit (Bio-Rad, Hercules, CA, USA). Proteins were resolved using SDS-polyacrylamide gel electrophoresis gels (Life Technologies, Carlsbad, CA, USA) and transferred onto polyvinylidene fluoride (PVDF) membranes (Millipore, Burlington, MA, USA). The primary antibodies used were anti-LL-37 (1:500), anti-IGFBP-2 (1:500), anti-IGFBP-3 (1:500), and anti-tubulin as an internal control, with detection achieved using horseradish peroxidase-conjugated secondary antibody (1:5000) and visualization with electrochemical luminescence (ECL) reagent (GE Healthcare, Chicago, IL, USA).

For the RT-qPCR assay, total RNA extraction was followed by cDNA synthesis using the SuperScript^®^ III RT kit (Invitrogen, Carlsbad, CA, USA). Cytokine, metalloproteinase, IGFBP-3, and LL-37 measurements in cell culture supernatants were performed using specific ELISA kits for IL-6, TNF-α, TGF-β, IL-10, MMP-1, MMP-3, and MMP-13 (R&D Systems, Minneapolis, MN, USA), according to the manufacturer’s instructions. Transwell chambers (Corning, Corning, NY, USA) were used for co-cultures, and all other chemicals were of analytical grade supplied by Sigma-Aldrich (St. Louis, MO, USA), unless otherwise indicated.

### 2.2. Cell Culture and Treatment

The cell line employed were the human keratinocyte cell line (Rio de Janeiro Cell Bank, Rio de Janeiro, RJ, Brazil) and CCD1072SK human fibroblasts cells (Rio de Janeiro Cell Bank). Human Keratinocytes (hKT) were cultured in Dulbecco Modified Eagle Medium (DMEM; Gibco, Carlsbad, CA, USA) containing 4 mM L-glutamine and 1.5 g/L sodium bicarbonate. The culture medium was supplemented with 10% fetal bovine serum, 100 μg/mL streptomycin, 100 μg/mL penicillin, and 0.1% gentamicin. The CCD1072SK human fibroblasts cells were cultivated in DMEM culture medium supplemented with 10% Fetal Bovine Serum and 0.1 mg/mL gentamicin at 37 °C in a humidified atmosphere with 5% CO_2_. Only confluent cells between the 3rd and 12th passage were harvested for further experiments. For establishment of co-cultures, CCD1072SK were plated at a density of 1.5 × 104 cells/cm^2^ in six-well plates (1.25 × 10^5^/well), and incubated in DMEM and 10% FCS for 24 h. The hKT cells were added, at a 1:6 ratio (keratinocytes/fibroblasts), yielding a density of 2.5 × 10^3^ keratinocytes/cm^2^, and allowed to attach overnight. For co-cultures with hKT, FAD medium containing 10% FCS was used. To physically separate keratinocytes from fibroblasts in co-cultures, hKT cells were seeded in the upper compartment of transwell chambers and plated fibroblasts in the lower compartment. This co-culture separation (Transwell) configuration was used for all subsequent functional assays, such as cytokines, MMPs, LL-37, IGFBP-3, and TGF-β, to evaluate paracrine effects through soluble mediators. Medium was subsequently replaced every 2 days. For the cytotoxicity assessment of Cleoderm, an MTT assay was performed where cells were treated with serial dilutions of the product in DMEM/0.5% FBS at concentrations of 0.001%, 0.01%, 0.1%, 1.0%, and 10% (*v/v*), using cells maintained in DMEM/0.5% FBS only as a control. A positive control, consisting of cells treated with 1% (*v/v*) Triton X-100, was also included in the assay to ensure the validity and maximum range of detection of the MTT assay. Based on the results of this assay, the concentrations of 1.0% and 10% (*v/v*) Cleoderm™ were selected for subsequent experiments. For the functional assays, the cultures were divided into five experimental groups: control (cells maintained in DMEM/0.5% FBS), LPS (cells stimulated with 1 µg/mL LPS for 24 h), LPS + Cleoderm™ 1.0% (cells pre-treated with Cleoderm™ 1.0% for 1 h, followed by 24 h of co-incubation with 1 µg/mL LPS), LPS + Cleoderm™ 10.0% (cells pre-treated with Cleoderm™ 10.0% for 1 h, followed by 24 h of co-incubation with 1 µg/mL LPS), and Cleoderm™ 10.0% (cells treated with the highest concentration of the product alone for 24 h to evaluate its intrinsic effects). After the respective treatments, cells were harvested for subsequent analysis.

### 2.3. Cytotoxicity Assays

For cytotoxicity assays, 1 × 10^4^ hKT and CCD1072SK cells/mL were seeded in 96-well plates. The cells were exposed for 24 h to the following concentrations of Cleoderm™: 0.01%, 0.1%; 1.0%; and 10%. The Trypan Blue assay was performed after different treatment protocols. To measure cell viability, control cells and samples treated with Cleoderm™ were centrifuged and suspended in equal volumes of medium and 0.05% trypan blue solution, and counted using a hemocytometer chamber. For the MTT assay, a sufficient volume of concentrated solution was added to achieve a final concentration of 0.04 mg per well. After a two-hour incubation, the medium was removed by inversion, and 200 µL of DMSO was added for crystal release.

### 2.4. Western Blot Analysis

Western blotting for evaluation of LL-37, and IGF-1 (IGFBP-2 and IGFBP-3) protein levels after exposure of co-culture to Cleoderm™. The Cleoderm™-treated cells were lysed in NP-40 lysis buffer (50 mmol/L Tris-HCl (pH 7.4), 1% NP-40, 150 mmol/L NaCl, 5 mmol/L EDTA, 50 mmol/L NaF, 30 mmol/L Na_4_P_2_O_7_, 1 mmol/L Na_3_VO_4_) supplemented with protease and phosphatase inhibitors (protease inhibitor cocktail plus 5 mmol/L sodium fluoride, 0.5 mmol/L sodium orthovanadate, 1 mmol/L sodium molybdate, 50 mmol/L 2-chloroacetamide, 2 mmol/L 1,10-phenanthroline monohydrate, and 0.5 mmol/L phenylmethanesulfonyl fluoride (Sigma). The lysates were incubated at 4 °C for 30 min in ice. After centrifugation at 4 °C for 10 min at 13,000 rpm/min in a 5.5 cm rotor radius microcentrifuge, the protein concentration was determined using a Bradford assay (Biorad, Hercules, CA, USA). From the total proteins, 50 lg was resolved using SDS–polyacrylamide gel electrophoresis gels (Life Technologies) and electroblotted onto polyvinylidene fluoride (Millipore) membranes. The blots were incubated with primary antibodies anti-LL-37 1:500 (AFG Bioscience, Wilmington, DE, USA); anti-TGF-β 1:500 (Elabscience, Wuhan, China); and anti-IGFBP-3 1:500 (AFG Bioscience), suspended in 5% nonfat dry milk in PBS plus 0.1% Tween-20 overnight at 4 °C. The detection was achieved using a horseradish peroxidase-conjugated secondary antibody (1:5000) in 5% nonfat dry milk in PBS plus 0.1% Tween-20, Jackson Immuno Research Laboratories, and visualized with electrochemical luminescence (ECL, GE Healthcare). The images were recorded using a Chemidoc apparatus (Uvitec, Cambridge, UK). The β-tubulin was used as an internal control. The specificity of the primary antibodies was confirmed by the detection of bands at the predicted molecular weight for each target. 

### 2.5. Reverse Transcription-Quantitative PCR (RT-qPCR)

Total RNA extracted from cell samples was converted to cDNA using a SuperScript^®^ III RT kit (Invitrogen, Carlsbad, CA, USA), according to the manufacturer’s protocol. The concentration of RNA was detected using a NanoDrop 2000 (Thermo Fisher Scientific, Inc., Waltham, MA, USA). GAPDH was used as an internal control, and its stability under experimental conditions was validated in a subset of samples against a second reference gene, *beta-actin*, confirming that GAPDH expression remained stable. The thermocycling conditions were as follows: 95 °C for 10 min followed by 35 cycles of 95 °C for 15 s and 55 °C for 40 s. The 2^−ΔΔCq^ method was used to quantify the relative gene expression levels of the target genes. Relative standard curves were generated by serial dilutions, and all samples were run in triplicate. The primers used for all targets were based on previously validated sequences (references), and their specificity and amplification efficiency (R^2^ > 0.98) were confirmed by dissociation and internal dilution curves prior to use, indicating no amplification of pseudogenes. Table 1 indicates the sense and antisense sequences of primers used in qRT-PCR analysis.

### 2.6. Cytokines Analysis in Cell Culture Cupernatants

The concentrations of IL-6, TNF-alfa, IGFBP3, TGF-β, IL-10, MMP-1, MMP-3, and MMP-13 in the cell co-culture supernatants were analyzed by using enzyme-linked immunosorbent assay (ELISA) kits (R&D Systems, Minneapolis, MN, USA) following the manufacturer’s instructions. Cells were pretreated with LPS (1 μg/mL) for 60 min with or without Cleoderm™ (1.0% and 10.0%) for 24 h. The cell culture supernatant (100 μL) was collected to determine the levels of proteins, according to the manufacturer’s instructions. To avoid interference from substances that could compromise sample clarity, the supernatants were centrifuged at 1500× *g* for 10 min at 4 °C prior to ELISA analysis in order to remove undissolved lipids and suspended particles.

### 2.7. Statistical Analysis

Results are shown as mean ± SEM from at least three separate experiments. Student’s *t*-test was used to analyze paired data. For comparisons among multiple groups, one-way ANOVA was employed. In all tests, a *p*-value below 0.05 was deemed significant. The normality of the data distribution was assumed based on visual inspection and previous experience with the biological models used. Homogeneity of variances was confirmed by Levene’s test prior to the one-way ANOVA.

## 3. Results

### 3.1. Cleoderm™ Exhibits No Cytotoxic Effects in Human Keratinocytes or Fibroblasts

**Figure 1:**
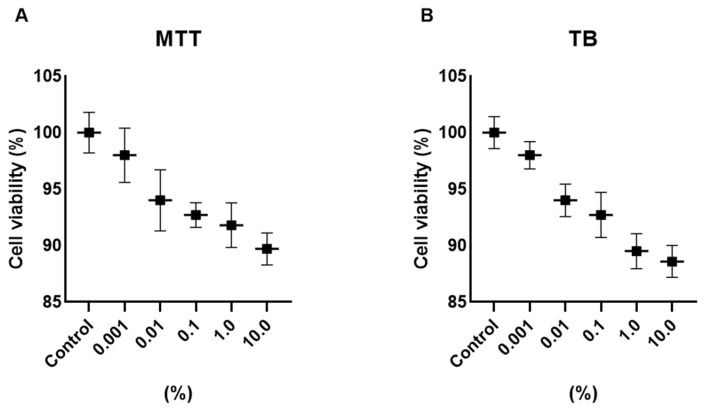
Determination of the viability of hKT cells after treatment with different concentrations of Cleoderm™ over a period of 24 h. The tests performed were MTT (**A**) and Trypan Blue (TB) (**B**). The values were not significantly different compared to the control group (ANOVA and Tukey post hoc test).

Cell viability was assessed using MTT and Trypan Blue exclusion assays after 24 h exposure to Cleoderm™ at concentrations ranging from 0.01% to 10.0% (*v/v*). Across all tested concentrations, no significant cytotoxicity was observed in either keratinocytes or fibroblasts compared with untreated controls (*p* > 0.05). Cell viability remained above 90% in all conditions, confirming the non-toxic nature of Cleoderm™ and validating the selected concentrations (1.0% and 10.0%) for subsequent experiments (Figure 1). The use of non-ionic, low-interfering surfactants in the formulation minimizes the risk of non-specific artifacts from vehicle components in the viability assays, supporting the biological relevance of the concentrations selected.

These results demonstrate that Cleoderm™ maintains cellular integrity and metabolic activity even at high concentrations, supporting its safety for topical and compounding use.

### 3.2. Cleoderm™ Modulates Key Regulatory Biomarkers: IGFBP-3, TGF-β, and LL-37

**Figure 2:**
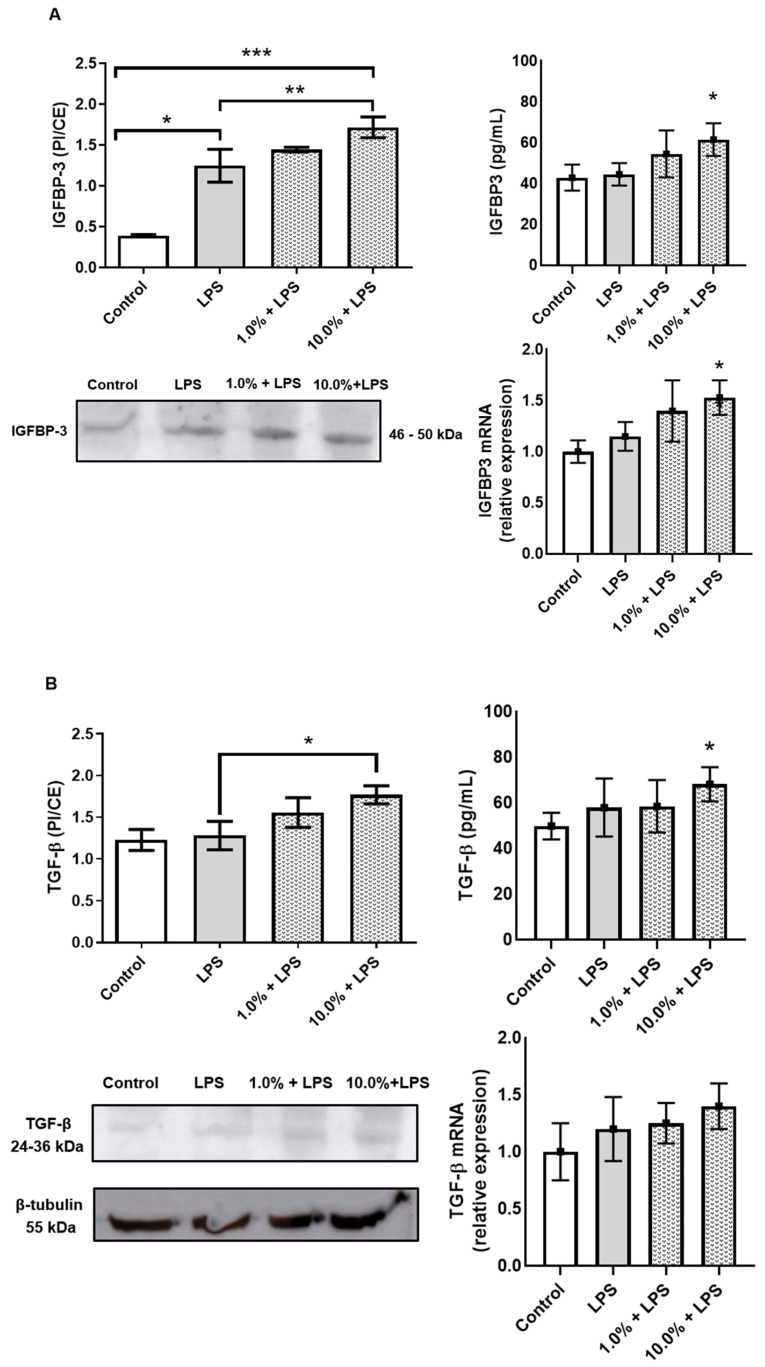
Modulation of IGFBP-3 and TGF-β protein, secretion, and mRNA levels by Cleoderm™ in hKT cells after 24 h of treatment. (**A**) shows IGFBP-3 expression, and (**B**) shows TGF-β expression. Protein levels were determined by Western blot (bands shown below) with densitometric quantification presented in the bar graphs. Secreted protein levels in the culture supernatants were determined by ELISA, and mRNA levels were quantified by Real-Time PCR. Data are presented as mean + SEM and are representative of three independent experiments (*n* = 3). Statistical differences were evaluated using ANOVA followed by Tukey’s post hoc test. (*) Values significantly different from the LPS-treated control group (*p* < 0.05). (**) Values significantly different from the LPS-treated control group (*p* < 0.01). (***) Values significantly different from the LPS-treated control group (*p* < 0.001).

**Figure 3:**
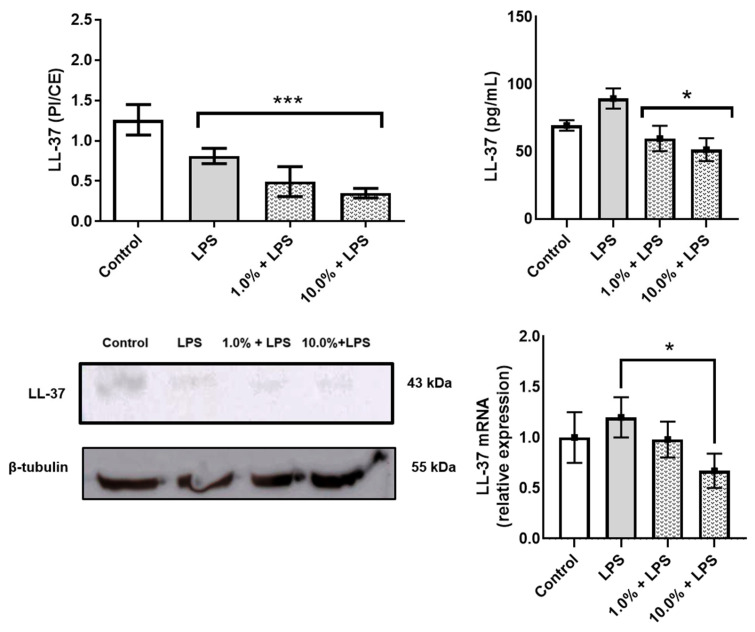
Modulation of LL-37 protein, secretion, and mRNA levels by Cleoderm™ in hKT cells after 24 h of treatment. Protein levels were determined by Western blot (bands shown below) with densitometric quantification presented in the bar graphs. Secreted protein levels in the culture supernatants were determined by ELISA, and mRNA levels were quantified by Real-Time PCR. Data are presented as mean + SEM and are representative of three independent experiments (*n* = 3). Statistical differences were evaluated using ANOVA followed by Tukey’s post hoc test. (*) Values significantly different from the LPS-treated control group (*p* < 0.05). (***) Values significantly different from the LPS-treated control group (*p* < 0.001).

Western blot and ELISA analyses revealed that Cleoderm™ significantly modulated several biomarkers implicated in sebaceous regulation, tissue repair, and innate immunity. Treatment with Cleoderm™ (1.0% and 10.0%) significantly increased IGFBP-3 protein expression compared with LPS-stimulated controls (*p* < 0.05; Figure 2A). This suggests modulation of the IGF-1 signaling axis, a pathway involved in sebocyte lipogenesis and keratinocyte proliferation, both central to acne pathophysiology. Similarly, Cleoderm™ induced a marked upregulation of TGF-β, a cytokine known for its anti-inflammatory and reparative functions (*p* < 0.05; Figure 2B). In contrast, Cleoderm™ significantly downregulated LL-37, an antimicrobial peptide and known pro-inflammatory mediator in rosacea. Both protein and mRNA levels of LL-37 were reduced after Cleoderm™ treatment (*p* < 0.05; Figure 3).

Together, these findings indicate that Cleoderm™ exerts a multifaceted immunomodulatory and reparative effect, targeting distinct yet interconnected biological axes relevant to inflammatory skin conditions.

### 3.3. Cleoderm™ Reduces Pro-Inflammatory Cytokine Expression and Enhances Anti-Inflammatory Response

**Figure 4:**
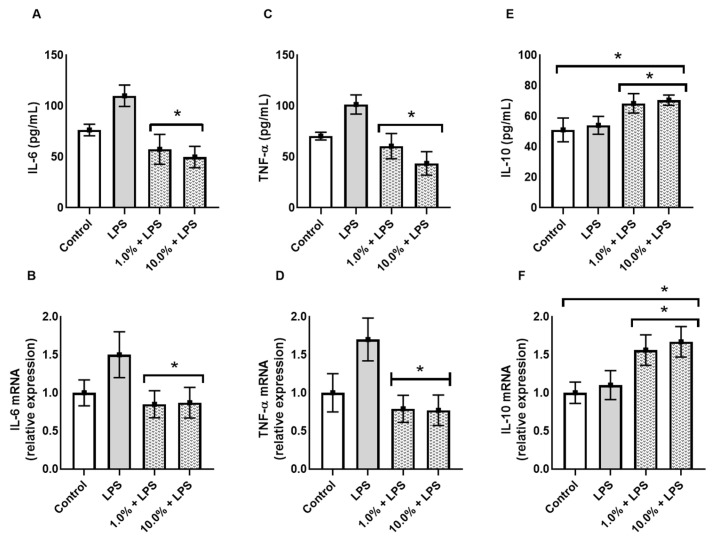
Cleoderm™ (1.0% and 10%) modulates the secretion and mRNA expression of inflammatory and regulatory cytokines after 24 h of treatment. (**A**,**B**) show IL-6 protein secretion and mRNA quantification, (**C**,**D**) show TNF-α protein secretion and mRNA quantification, and (**E**,**F**) show IL-10 secretion and mRNA quantification. Data are expressed as mean + SEM. (*) Statistical significance versus the LPS-treated control group was determined by one-way ANOVA with Tukey’s post hoc test (*p* < 0.05).

To evaluate the immunomodulatory potential of Cleoderm™, the secretion and gene expression of key cytokines were quantified following LPS stimulation. As shown in Figure 4, exposure to LPS significantly upregulated IL-6 and TNF-α expression in the co-culture model, confirming successful induction of an inflammatory state.

Treatment with Cleoderm™ 10.0% significantly reduced LPS-induced IL-6 and TNF-α secretion. The 10.0% concentration resulted in an approximately 43% reduction in IL-6 secretion (~115 pg/mL vs. ~65 pg/mL; Figure 4A; *p* < 0.05), and mRNA was reduced by approximately 44% (Figure 4B; *p* < 0.05). More markedly, TNF-α secretion was reduced by approximately 50% (Figure 4C; *p* < 0.05), accompanied by a greater than 55% reduction in mRNA expression (Figure 4D; *p* < 0.05). Conversely, Cleoderm™ significantly upregulated IL-10, an anti-inflammatory cytokine associated with tissue protection and immune regulation (*p* < 0.05). In contrast to pro-inflammatory cytokines, Cleoderm™ 10.0% demonstrated a positive modulation of the regulatory cytokine IL-10. Treatment resulted in a significant increase of approximately 40% in IL-10 protein secretion (from 50 pg/mL in LPS to 70 pg/mL in LPS + 10.0%; Figure 4E; *p* < 0.05). This effect was corroborated by mRNA quantification, which showed an increase of approximately 70% in relative expression (1.7 times greater) compared to the LPS group (Figure 4F; *p* < 0.05). This induction of IL-10 suggests an active counter-inflammatory regulatory pathway by the formulation.

This dual modulation—downregulation of pro-inflammatory and upregulation of anti-inflammatory mediators—indicates a potent immunoregulatory effect of Cleoderm™, consistent with its hypothesized role as a dermofunctional base with intrinsic bioactivity.

### 3.4. Cleoderm™ Inhibits Matrix-Degrading Enzymes (MMP-1, MMP-3, and MMP-13)

**Figure 5:**
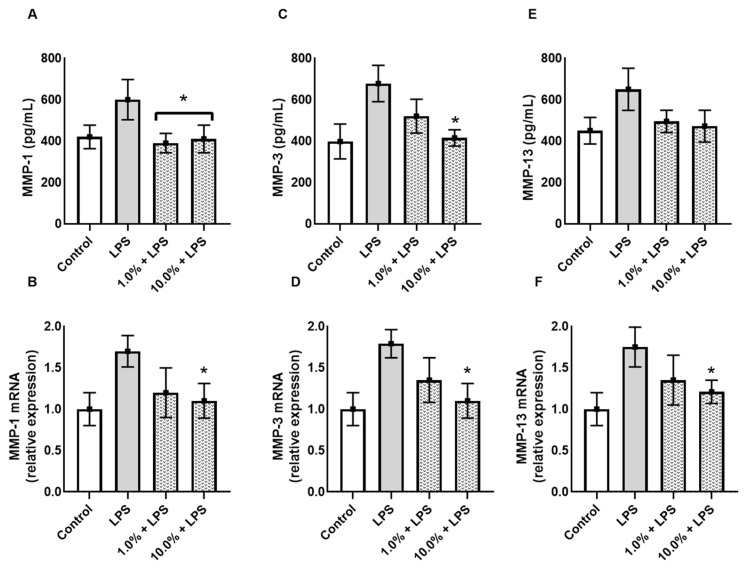
Effect of Cleoderm™ (1.0% and 10%) on the secretion and mRNA quantification of matrix metalloproteinases in hKT cells after 24 h of treatment. (**A**,**B**) show MMP-1 secretion and mRNA levels; (**C**,**D**) show MMP-3 secretion and mRNA levels; and (**E**,**F**) show MMP-13 secretion and mRNA levels. Data are presented as mean + SEM. Statistical differences were evaluated by ANOVA followed by Tukey’s post hoc test. (*) Values significantly different from the LPS group (*p* < 0.05).

To assess the impact of Cleoderm™ on extracellular matrix (ECM) integrity, levels of matrix metalloproteinases (MMPs) were analyzed. LPS stimulation led to a significant increase in MMP-1, MMP-3, and MMP-13 secretion and mRNA expression, consistent with an inflammatory and tissue-degrading environment (Figure 5). Cleoderm™ treatment at both tested concentrations significantly reduced MMP-1, MMP-3, and MMP-13 at both transcript and protein levels (*p* < 0.05). These results suggest that Cleoderm™ exerts matrix-protective properties, likely by suppressing inflammatory signaling cascades that drive MMP induction. Such inhibition of matrix-degrading enzymes is relevant to dermatoses like acne and rosacea, in which ECM breakdown contributes to lesion persistence, erythema, and scarring.

## 4. Discussion

In a physiologically oriented keratinocyte–fibroblast co-culture, Cleoderm™ demonstrated intrinsic bioactivity that aligns with key pathogenic nodes in common inflammatory dermatoses. Across all assays, Cleoderm™ reduced pro-inflammatory cytokines (IL-6, TNF-α), suppressed matrix-degrading enzymes (MMP-1, -3, -13), attenuated LL-37, and increased anti-inflammatory and reparative mediators (IL-10, TGF-β) together with IGFBP-3. These convergent effects, observed in this in vitro dermo-epidermal model, support the concept of Cleoderm™ as an active vehicle capable of favorably modulating cutaneous homeostasis while serving as a delivery base for customized formulations.

The LPS-stimulated co-culture model was used at this initial stage as a strategic tool to evaluate the core anti-inflammatory bioactivity of Cleoderm™. While it does not replicate the full complexity of acne or rosacea, it allowed us to isolate and demonstrate Cleoderm™’s ability to modulate central inflammatory pathways, such as the expression of LL-37 and IGFBP-3, which are relevant to these dermatoses. The results validate this approach as an informative starting point and guide the next steps of research, which will include specific models using *C. acnes*, sebocytes, and neuroimmune stimuli to confirm clinical relevance.

Another aspect of the study concerns the absence of an inert vehicle or standard cream control. The experimental design, which included medium and LPS-only controls, was deliberately structured to prioritize the direct assessment of Cleoderm™ intrinsic bioactivity and its capacity to modulate pathogenic pathways under basal and inflammatory conditions. Within this framework, the study focused on identifying formulation-specific biological responses rather than general vehicle-related effects. While the inclusion of a vehicle control could provide an additional level of comparative refinement, the magnitude and specificity of the observed effects, particularly the robust modulation of key biomarkers such as LL-37 and IGFBP-3, suggest the presence of an active and targeted mechanism that extends beyond the expected contribution of an inert vehicle. To further consolidate this positioning and enhance the comparative robustness of the model, future studies will incorporate a composition-matched vehicle control devoid of active ingredients, as suggested.

From a methodological perspective, it is important to note that the 10.0% concentration of Cleoderm™ used in the functional assays is relatively high when applied directly to an in vitro cell model. However, it is crucial to note that this concentration was selected based on a preliminary cytotoxicity assay (Figure 1), in which cell viability remained above 90%, allowing us to infer that the observed effects do not result from osmotic, mechanical, or general cytotoxicity stress. Furthermore, the specificity and duality of the biological responses, notably the significant upregulation of IGFBP-3 and the downregulation of LL-37 and pro-inflammatory cytokines, are consistent with a targeted biochemical mechanism of action, and not with a nonspecific effect associated with a high vehicle concentration. Thus, this concentration was employed as a maximum robustness test to isolate the intrinsic bioactivity of the formulation. Although MTT viability was not reduced, it is recognized that MTT is limited and does not rule out subtle metabolic suppression.

The most salient outcome of this work is the dual modulation of LL-37 and IGFBP-3. Cleoderm™ produced a clear reduction in LL-37 by Western blot and an even greater decrease in its secreted levels by ELISA, which we consider central to its mechanism of action. The divergence between the modest intracellular change and the pronounced suppression of secreted LL-37 is mechanistically informative: it suggests Cleoderm™ may influence not only synthesis but also processing, storage, or active secretion of LL-37. This is clinically pertinent because the pathogenic activity of LL-37 in rosacea and acne is predominantly mediated by its extracellular, processed forms, which amplify inflammation via TLR pathways [28,29]. However, since the study focused on quantifying the final peptide and did not include measurements of hCAP18 or kallikrein 5 (KLK5) activity, it was not possible to determine whether the observed reduction resulted from decreased precursor synthesis or negative modulation of processing proteases.

Concurrently, Cleoderm™ upregulated IGFBP-3, pointing to a targeted effect on the IGF-1 axis [30]. In acne, IGF-1 signaling promotes sebaceous lipogenesis and inflammatory drive [18,19,30]. By increasing IGFBP-3, an IGF-1 high-affinity binding protein, the bioavailable fraction of IGF-1 that can engage its receptor is effectively curtailed, limiting downstream sebotropic and pro-inflammatory signaling [31]. This buffering mechanism supplies a molecular rationale for anti-acne activity that extends beyond sebum reduction per se (ascribed to *C. gynandra* data) toward normalization of the hormonal signaling that governs sebaceous function [30]. It is worth noting that the findings obtained in this study is consistent with the mechanistic hypothesis of Cleoderm™; however, studies evaluating the measurement and activity of IGF-1 and other pathways related to acne in in vitro sebocyte models and lipogenesis assays should be conducted.

The immune milieu also shifted toward resolution and repair, evidenced by the induction of IL-10 and TGF-β. In line with this, the strong anti-inflammatory signal observed, namely the marked reductions in IL-6 and TNF-α, is plausibly a downstream consequence of LL-37 attenuation [22,32]. Elevated IL-10 is consistent with suppression of macrophage activation and pro-inflammatory cytokine release [33], whereas increased TGF-β supports tissue repair and re-establishment of epidermal barrier function [34]. Although the quantification of TGF-β suggests a role in the action of Cleoderm™, it will be necessary in a future step to verify the activation of this protein in order to assess its impact on barrier repair. These changes align with prior reports implicating *Cleome gynandra* extracts and palmitoyl tripeptide-8 as modulators of cutaneous inflammation and stress responses [35,36,37]. The suppression of pro-inflammatory cytokines, such as IL-6 and TNF-α, suggests that Cleoderm™ may act by inhibiting the cellular stress signaling pathway. However, it is crucial to recognize that this study focused on screening biomarkers such as cytokines and MMPs; downstream mechanistic assays, such as analysis of NF-kappa B nuclear translocation or phosphorylation of p38 and other stress kinases, were not performed. Therefore, confirmation of the specific molecular pathway of action of Cleoderm™ remains the logical and necessary next step to solidify the inferred mechanism.

This anti-inflammatory, pro-reparative context translated into tissue protection at the structural level. Cleoderm™ downregulated MMP-1, MMP-3, and MMP-13, reinforcing preservation of the extracellular matrix, a crucial objective in chronic inflammatory dermatoses [24]. Because MMP overexpression, often driven by LL-37 and cytokines, accelerates collagen degradation and dermal remodeling [38], its suppression supports matrix homeostasis and could improve texture and resilience [39]. Notably, lowering these MMPs indicates Cleoderm™ not only tempers inflammation but may also limit downstream remodeling processes that contribute to scarring and telangiectasias [24]. Thus, the most likely hypothesis for how Cleoderm™ acts in relation to MMPs is through reduction via upstream inflammatory signaling of TNF-α and IL-6, although future studies of MMP promoter activity are needed to confirm direct inhibition.

Mechanistically, the multi-target profile can be traced to Cleoderm’s composition. The patented *Cleome gynandra* extract is a strong candidate for dampening LL-37 and TNF-α via TLR2 inhibition [35] and by mitigating oxidative stress that fuels inflammatory amplification [36]. Palmitoyl tripeptide-8, a neuro-soothing peptide [28], together with bisabolol—a well-known anti-inflammatory and permeation enhancer [11]—likely synergizes to attenuate neurogenic and cytokine-driven inflammation. Meanwhile, the functional oils and hyaluronic acid contribute to barrier repair and hydration, fostering a cutaneous environment less susceptible to inflammatory triggers [40].

In summary, Cleoderm™ acts through a coordinated, multi-component mechanism: (i) it targets root inflammatory drivers by modulating LL-37 and the IGF-1/IGFBP-3 axis; (ii) it promotes a resolution phenotype via IL-10 and TGF-β; (iii) it controls acute cytokine activity by reducing IL-6 and TNF-α; and (iv) it preserves tissue integrity by suppressing destructive MMPs. Together, these effects indicate that Cleoderm™ functions not as an inert carrier but as an active participant in restoring cutaneous homeostasis, supporting its role as a functional vehicle capable of enhancing the performance of incorporated actives.

### Limitations and Future Directions

The inherent limitations of the study should guide the interpretation of our results. Thus, the data are derived from a two-dimensional in vitro monoculture model, which limits the complex pathophysiology of acne. Furthermore, this is an exploratory study focused on biomarker screening, not including in vivo data and still requiring further downstream mechanistic assays. Although our in vitro model provided important information on keratinocyte and fibroblast responses, future studies incorporating sebocytes may offer a more comprehensive view of the complex pathophysiology of acne. Furthermore, the potential interactions between the formulation and the skin microbiome remain an area requiring further investigation.

## 5. Conclusions

Cleoderm™ demonstrated intrinsic and multi-axis bioactivity in a relevant dermo-epidermal co-culture model, characterized by the suppression of pro-inflammatory mediators (IL-6, TNF-α), the down-regulation of LL-37, the inhibition of matrix-degrading MMP-1/-3/-13, and the concomitant up-regulation of reparative cytokines (IL-10 and TGF-β). Notably, the induction of IGFBP-3 points to the modulation of the IGF-1 pathway, offering a possible mechanistic basis for anti-acne benefits that extend beyond sebum reduction alone. It is important to emphasize that, although this co-culture dataset provides coherent and conceptually compelling evidence, particularly focusing on LL-37 and IGFBP-3 as dermofunctional readouts, the inherent limitations of in vitro models require caution in directly transposing mechanisms and clinical benefits. Further in vivo studies are needed to fully validate the scope of action and clinical relevance of Cleoderm™ in topical formulations. Taken together, these effects support the positioning of Cleoderm™ as an active dermofunctional base rather than an inert vehicle, with the potential to improve the efficacy and tolerability of the active ingredients incorporated in customized formulations.

## Figures and Tables

**Table 1:** Forward and reverse primer sequences used for RT-qPCR.

*Gene*	Forward (5′→3′)	Reverse (5′→3′)	Ref.
*GAPDH*	CGT CTT CAC CAC CAT GGA GA	CGG CCA TCA CGC CAC AGT TT	[22]
*IL-6*	GTG TGA AAG CAG CAA AGA GGC	CTG GAG GTA CTC TAG GTA TAC	[22]
*TNF-α*	CAA AGT AGA CCT GCC CAG AC	GAC CTC TCT CTA ATC AGC CC	[22]
*IL-10*	AAG GCA GTG GAG CAG GTG AA	CCA GCA GAC TCA ATA CAC AC	[23]
*MMP-1*	GGC CCA CAA ACC CCA AAA G	ATC TCT GTG GGC AAA TTC GTA AGC	[24]
*MMP-3*	GAT GCC CAC TTT GAT GAT GAA	AGT GTT GGC TGA GTG AAA GAG ACC	[24]
*MMP-13*	GGC CCA ACC CTA AAC ATC CAA AAA C	TTA AAA ACA GCT CCG CAT CAA CCT	[24]
*TGF-β*	ACC GCA ACA ACG CCA TCT AT	GTA ACG CCA GGA ATT GTT GC	[25]
*LL-37*	AGG ATT GTG ACT TCA AGA AGG ACG	GTT TAT TTC TCA GAG CCC AGA AGC	[26]
*IGFBP3*	ATG CAG CGG GCG CGA C	CTA CTT GCT CTG CAT GCT GTA GCA	[27]

## Data Availability

The original contributions presented in this study are included in the article. Further inquiries can be directed to the corresponding author.

## Abbreviations

The following abbreviations are used in this manuscript:

DMEM	Dulbecco’s Modified Eagle Medium
FBS	Fetal Bovine Serum
LPS	Lipopolysaccharide
MTT	3-(4,5-dimethylthiazol-2-yl)-2,5-diphenyltetrazolium bromide
DMSO	Dimethyl sulfoxide
ELISA	Enzyme-linked immunosorbent assay
RT-qPCR	Reverse transcription-quantitative PCR
MMP	Matrix metalloproteinase
IGFBP-3	Insulin-like growth factor-binding protein 3
TGF-β	Transforming growth factor beta
IL	Interleukin
TNF-α	Tumor necrosis factor alpha
ECM	Extracellular matrix
PVDF	Polyvinylidene fluoride
ECL	Electrochemical luminescence
SDS-PAGE	Sodium dodecyl sulfate–polyacrylamide gel electrophoresis
